# Influence of Crown Height and Width on Marginal Bone Loss and Long-Term Stability of Dental Implants: A Systematic Review

**DOI:** 10.7759/cureus.65109

**Published:** 2024-07-22

**Authors:** Ritul Jain, Sweta G Pisulkar, Surekha A Dubey, Akansha Bansod, Arushi Beri, Shruti Deshmukh

**Affiliations:** 1 Department of Prosthodontics and Crown and Bridge, Sharad Pawar Dental College and Hospital, Datta Meghe Institute of Higher Education & Research, Wardha, IND

**Keywords:** bone density, short implants, implant stability, crown-implant ratio, dental implants, marginal bone loss, crown width, crown height

## Abstract

Still, a major focus of research in implantology is how crown height and width affect marginal bone loss (MBL) and the long-term durability of dental implants. Maximizing the success of implants and lowering problems depends on an awareness of these elements. Following the Preferred Reporting Items for Systematic reviews and Meta-Analyses (PRISMA) guidelines, this systematic review searched pertinent studies across several databases using keywords unique to databases. Studies on MBL and long-term implant stability evaluated in the review included those on crown height and width, horizontal and vertical cantilevers, and prosthesis dimensions. In the chosen studies, we found that both implant success and crestal bone loss were greatly influenced by crown height and width. Particularly in the posterior sections, horizontal cantilevers were connected to both increasing MBL and mechanical problems. Vertical cantilevers also affected MBL; however, their impacts were more obvious in circumstances with greater crown heights. Greater prosthesis widths, especially in the mandibular molar area, were linked to higher MBL. Bone density and insertion torque (IT) were the main determinants of MBL, more than the primary implant stability quotient. Early MBL was influenced by abutment height, mucosal thickness, and implant insertion depth; bone levels stabilized six months later. Short implants allow single crowns to be supported, but in some cases, a higher failure rate was seen. The success and stability of dental implants were found to be mostly dependent on crown height, width, and cantilever design. MBL and long-term stability are greatly influenced by horizontal and vertical cantilevers, which calls for careful design and planning. With specific care for bone density, IT, and early MBL stabilization, both short and standard implants can produce equivalent results. These results highlight the need for customized treatment plans to maximize implant success and lifetime.

## Introduction and background

Since they greatly enhance appearance and utility, dental implants are a necessary operation for restoring missing teeth in contemporary dentistry. The long-term health and performance of dental implants depend on maintaining the integrity of the nearby bone tissue, sometimes known as “peri-implant bone.” Marginal bone loss (MBL) is one crucial element influencing the success of dental implants. It is a common complication that, left ignored, can cause implant failure [1,2]. A complex problem affected by several biological and mechanical factors is bone loss surrounding dental implants [3]. Among the elements influencing the effectiveness of dental implants are the surgical method, implant design, patient-specific considerations, including bone quality and oral care practices, and prosthetic-related concerns [4]. Among all these factors, the design of the prosthetic crown, its width, and its height attracted the most interest since they may affect the biomechanics of the implant system [5,6].

The crown height, the vertical distance from the implant platform to the prosthetic crown’s occlusal surface, and the crown width, the horizontal dimension at the widest part of the crown, are two crucial factors that can affect how occlusal forces and stress are distributed at the bone-implant interface [7,8]. Biomechanical research suggests that an overly tall crown could cause concentrated stress and increased bending forces on the top of the tooth, therefore hastening bone loss. Conversely, the width of the crown might affect the distribution of stress across the implant, therefore influencing the pace of MBL [9] and the peri-implant bone response.

Notwithstanding great progress in implant technology and a lot of studies, our knowledge of the link between MBL and crown height and width is still lacking and occasionally conflicting. Higher rates of MBL and wider or taller crowns have been directly correlated in several studies [10]. Other research, however, has indicated that other elements might have a greater influence or have not found any clear association [9-11]. To maximize implant prosthetic designs and improve clinical outcomes, one must first fully understand how the width and height of the crown affect MBL. Therefore, the aim of this systematic analysis is to provide a full knowledge of the ways in which these prosthetic parameters affect implant outcomes and to shed light on the impacts of cantilever designs, crown width, and height on MBL and long-term implant stability.

## Review

Eligibility criteria

We followed the Preferred Reporting Items for Systematic reviews and Meta-Analyses (PRISMA) guidelines [12] to offer an open and all-encompassing reporting style. As explained below, the Population, Exposure, Comparator, Outcome (PECO) methodology provided a standardized structure for this systematic review, therefore providing a focused and methodical approach to the study topic. Patients who had undergone dental implant procedures made up the population of interest for this investigation. The study concentrated especially on the prosthetic crown height and width of these dental implants. Although there were several dental implants with varying crown height and width, the exploratory nature of the review made a precise comparator obsolete. The degree of bone loss noted around the dental implants was the major result of interest for this investigation.

Table 1 lists the several inclusion and exclusion rules developed for this review. The inclusion criteria were as follows: studies with a study design of randomized controlled trials (RCTs), cohort studies, case-control studies, and cross-sectional studies were included; studies that examined human participants who received dental implants, regardless of age, gender, or health status, were included; studies that provided specific measurements or descriptions of crown height and width of dental implants were included; studies that reported MBL around dental implants, measured through radiographic evaluations or clinical methods, were included; and articles published in English were included. On the other hand, the exclusion criteria were as follows: case reports, reviews, editorials, letters to the editor, and conference abstracts were excluded; animal models and in vitro experiments were excluded; studies without specific measurements or descriptions of crown height and width, or those assessing other prosthetic factors, were excluded; studies that did not report MBL or used non-standardized measurement methods were excluded; and articles published in languages other than English were excluded.

**Table 1:** Criteria for selection designed for this study MBL, marginal bone loss; RCT, randomized controlled trial

Criterion	Inclusion	Exclusion
Study design	RCTs, cohort studies, case-control studies, and cross-sectional studies	Case reports, reviews, editorials, letters to the editor, and conference abstracts
Population	Human participants who received dental implants, regardless of age, gender, or health status	Animal models, in vitro experiments
Exposure	Studies with specific measurements or descriptions of the crown height and width of dental implants	Studies without specific measurements or descriptions of crown height and width, or those assessing other prosthetic factors
Outcome	MBL around dental implants, measured through radiographic evaluations or clinical methods	Studies not reporting MBL or using non-standardized measurement methods
Language	Articles published in English	Articles published in languages other than English
Publication date	No restrictions on the publication date	-

Strategy for the selection of studies

Six separate databases - PubMed, Scopus, Cochrane Library, Web of Science, Embase, and Google Scholar - were searched using Boolean expressions and Medical Subject Headings (MeSH) keywords to improve the retrieval of pertinent material. The MeSH keywords used in the search strategy included “Dental Implants,” “Crown Height,” “Crown Width,” and “Marginal Bone Loss.” Additionally, MeSH phrases such as “Dental Implants AND Crown Height,” “Dental Implants AND Crown Width,” “Marginal Bone Loss AND Dental Implants,” “Crown Height AND Marginal Bone Loss,” and “Crown Width AND Marginal Bone Loss” were also utilized to capture relevant studies.

Data extraction technique

Using a consistent form, the data extraction procedure gathered pertinent information, therefore reducing bias by means of dual independent reviewers and resolving conflicts with a third reviewer. Study identification information (title, authors, year, and journal), study design (type, sample size, and follow-up length), and participant characteristics (age, gender, health status, and inclusion/exclusion criteria) comprised the form. Together with anatomical location and implant system type, key data, including crown height and width of dental implants, were painstakingly documented. The study concentrated on how horizontal and vertical cantilevers on implants affect MBL and long-term survival. This covered specifics on the vertical extension and crown height of vertical cantilevers, as well as their length and direction.

Method for bias assessment

This systematic review’s bias assessment method was devised to guarantee a thorough analysis of the included research. For this aim, two instruments were used: the ROBINS-I tool [13] and the RoB 2.0 tool [14].

Study selection schematics

**Figure 1:**
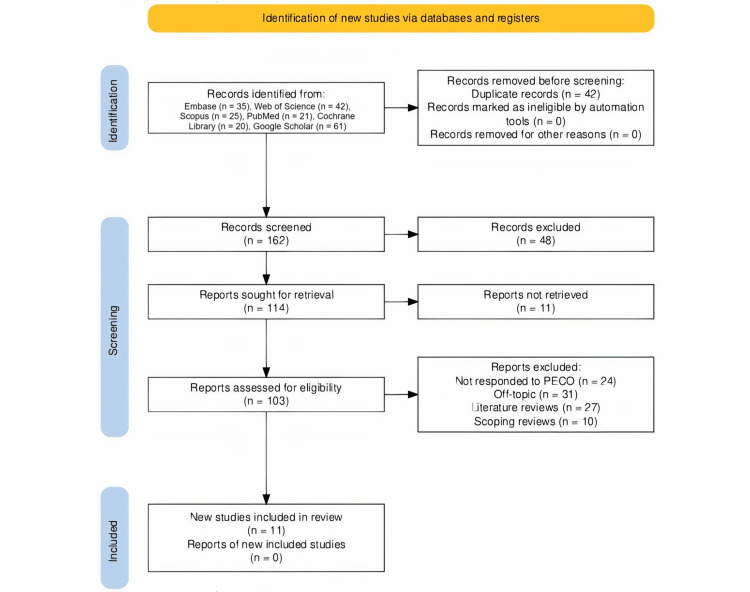
Article selection process for the review

The initial phase in the process of selecting articles for this study was the identification of 204 entries of records from several databases (Figure 1). Before screening, 42 duplicate records were removed, and no records were marked as ineligible by automation tools or removed for other reasons. A total of 162 records were screened, and 114 reports were sought for retrieval. Of these, 48 records were excluded, and 11 reports were not retrieved. The remaining 103 reports were assessed for eligibility, and 24 were excluded because they did not respond to the PECO criteria; 31 were off-topic; 27 were literature reviews; and 10 were scoping reviews. Eleven trials [15-25] were included in the final review, following which 151 publications were investigated for their eligibility.

Evaluated degrees of bias

**Figure 2:**
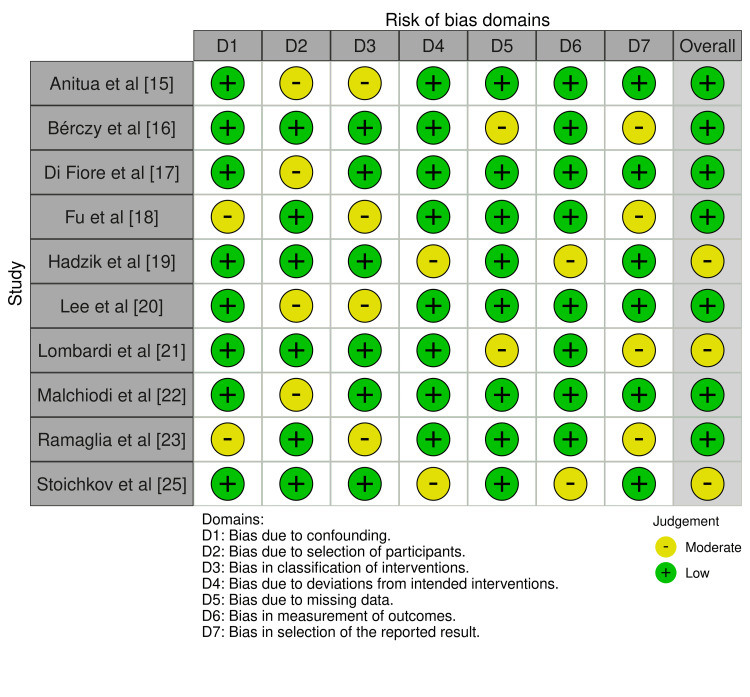
Bias evaluation using the ROBINS-I tool

The studies assessed using ROBINS-I (Figure 2) turned out to have a low or modest risk of bias overall. Many studies showed low general danger; only modest intermediate risk exists in particular fields. A few studies, nevertheless, showed modest overall risk because of moderate dangers in some areas.

**Figure 3:**
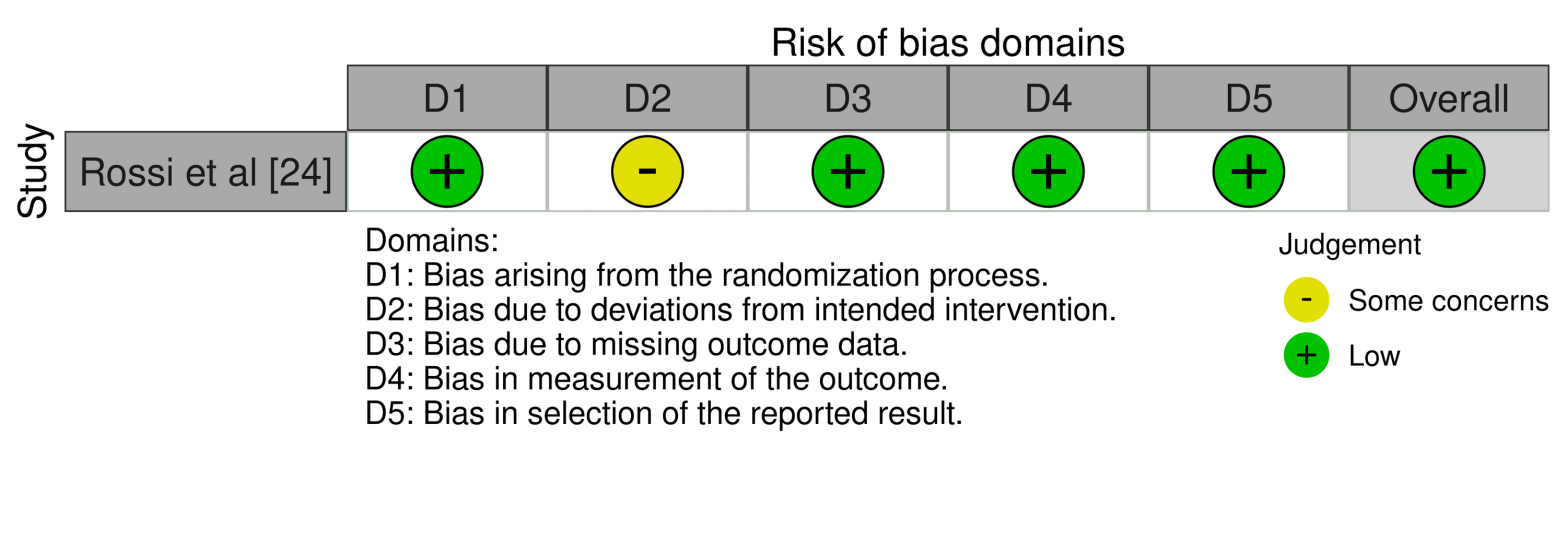
Bias evaluation using the RoB 2.0 tool

One study [24] assessed using the RoB 2.0 tool (Figure 3) showed a generally low likelihood of bias, however, with some particular reservations noted.

Sample sizes and study designs observed

Table 2 lists the included trials together with their corresponding observations [15-25]. Anitua et al. [15] performed a prospective cohort study comprising 136 patients and 259 implants, therefore allowing the gathering of thorough long-term data. With 34 patients and 60 implants, Bérczy et al. [16] investigated preexisting data using a retroactive cohort method to identify trends. Di Fiore et al. [17] also conducted a retrospective study with 65 patients and 65 implants, with an eye toward historical data. Fu et al. [18] adopted a prospective trial approach allowing for real-time data collection with 156 implants and 90 patients. Hadzik et al. [19] conducted a prospective experiment with 30 patients and 30 implants, therefore allowing real-time data collection. Lee et al. [20] performed a retroactive analysis including information on long-term outcomes using 259 implants and 175 patients.

**Table 2:** Included trials and their observed inferences C/I, crown-to-implant; ISQ, implant stability quotient; IT, insertion torque; MBL, marginal bone loss; PBL, peri-implant bone loss; RCT, randomized controlled trial; SPS, surface-modified platform switching

Author ID	Sample size	Study design	Implant characteristics (prosthesis height and width influence)	Horizontal and vertical cantilever characteristics	MBL observed	Long-term impact	Overall inference drawn
Anitua et al. [15]	136 patients, 259 implants	Prospective cohort	Prosthesis height varied from 8 mm to 12 mm, and width from 5 mm to 8 mm. Larger prosthesis dimensions linked to increased MBL.	Horizontal cantilevers up to 3 mm increased MBL; vertical cantilevers up to 2 mm had minimal impact.	Mean PBL: 0.48 ± 0.29 mm, significant correlation with C/I ratio (P < 0.05)	98.1% success rate, critical C/I ratio thresholds: 3.10 (anatomical) and 3.40 (clinical)	SPS implants show predictable success; the C/I ratio is a critical factor for implant success and crestal bone loss
Bérczy et al. [16]	34 patients, 60 implants	Retrospective cohort	Prosthesis height for short implants averaged 10 mm, and width 6 mm; standard implants had similar dimensions.	Horizontal cantilevers up to 3 mm for short implants showed increased MBL; vertical cantilevers had minimal impact.	Short implants: 1.2 ± 1.21 mm (mesial), 1.36 ± 1.47 mm (distal); Standard implants: 0.63 ± 0.80 mm (mesial), 0.78 ± 0.70 mm (distal)	Comparable success rates between short and standard implants	Short implants perform comparably to standard implants in terms of long-term success and MBL
Di Fiore et al. [17]	65 patients, 65 implants	Retrospective study	Crown height ranged from 10 mm to 14 mm, width from 5 mm to 8 mm. Higher crown heights linked to increased MBL.	Horizontal cantilevers up to 4 mm significantly increased MBL; vertical cantilevers up to 3 mm moderately increased MBL.	Higher C/I ratio (>1.5/1) linked to increased crestal resorption	Positive linear correlation between C/I ratio and crestal resorption	Higher C/I ratio in mandibular molars increases MBL compared to lower C/I ratios
Fu et al. [18]	90 patients, 156 implants	Prospective study	Prosthesis height averaged 11 mm, width 6 mm. Larger dimensions correlated with higher MBL.	Horizontal cantilevers up to 3 mm showed moderate MBL increase; vertical cantilevers up to 2 mm had minimal impact.	Significant impact of bone density and IT on MBL	Bone density and IT are more impactful on MBL than primary ISQ	Bone density and IT are critical for determining MBL, more so than primary ISQ
Hadzik et al. [19]	30 patients, 30 implants	Prospective study	Short implants had a prosthesis height of 9 mm, and a width of 5 mm; standard implants had a height of 11 mm and a width of 6 mm.	Horizontal cantilevers up to 3 mm for both implant types showed increased MBL; vertical cantilevers up to 2 mm had minimal impact.	Short implants: 0.34 ± 0.24 mm; Regular implants: 0.22 ± 0.46 mm	No significant correlation between the C/I ratio and stability or MBL	Short implants are viable for supporting single crowns; no significant difference in MBL or stability compared to regular implants
Lee et al. [20]	175 patients, 259 implants	Retrospective study	Prosthesis height ranged from 9 mm to 13 mm, width from 5 mm to 7 mm. Higher dimensions generally led to less MBL.	Horizontal cantilevers up to 3 mm had less impact on MBL; vertical cantilevers up to 2 mm showed minimal impact.	C/I ratio ≤1: greater MBL; C/I ratio >1: less MBL	The C/I ratio had a dominant influence on MBL, especially in the maxilla	Higher C/I ratio (>1) results in less MBL; site-related factors also impact MBL
Lombardi et al. [21]	50 patients, 83 implants	Multicenter prospective study	Prosthesis height varied from 8 mm to 12 mm, width from 4 mm to 6 mm. Early MBL was influenced by larger dimensions.	Horizontal cantilevers up to 3 mm increased early MBL; vertical cantilevers up to 2 mm had minimal impact.	Mean MBL: 1.24 ± 0.57 mm initially, 0.46 ± 0.59 mm at 12 months	Early MBL influenced by implant depth, mucosal thickness, and abutment height; stabilization after 6 months	Insertion depth, thin mucosa, and short abutments increase early MBL; bone levels stabilize after 6 months
Malchiodi et al. [22]	136 patients, 259 implants	Prospective cohort	SPS implants with a prosthesis height of 10 mm and, width of 6 mm. Larger dimensions are linked to increased MBL.	Horizontal cantilevers up to 3 mm increased MBL; vertical cantilevers up to 2 mm had minimal impact.	Mean PBL: 0.48 ± 0.29 mm, significant correlation with C/I ratio (P < 0.05)	98.1% success rate, C/I ratio thresholds: 3.10 (anatomical), 3.40 (clinical)	SPS implants are reliable for prosthetic rehabilitation; the C/I ratio is crucial for implant success and crestal bone loss
Ramaglia et al. [23]	78 implants in 34 mandibles and 44 maxillae	Longitudinal cohort	Short implants had a prosthesis height of 9 mm, and a width of 4 mm. Larger prosthesis dimensions were linked to increased MBL.	Horizontal cantilevers up to 3 mm increased MBL; vertical cantilevers up to 2 mm had minimal impact.	MBL < 0.5 mm, more pronounced in implants ≥10 mm and lower C/I ratios	No implant loss observed; bone loss associated with implant length and CIR	Higher CIR values do not increase peri-implant bone loss; short implants can be safely used for reduced bone heights
Rossi et al. [24]	45 patients, 60 implants	Prospective RCT	Short implants had a prosthesis height of 8 mm, and a width of 5 mm; standard implants had a height of 10 mm and a width of 6 mm. Larger dimensions led to increased MBL.	Horizontal cantilevers up to 3 mm for short implants showed increased MBL; vertical cantilevers up to 2 mm had minimal impact.	Higher MBL in short implants: five-year survival rates: 86.7% (6 mm) vs. 96.7% (10 mm)	Small MBL over five years, higher loss in short implants due to bone fracturing	Short implants show similar MBL to longer implants but a higher failure rate; suitable for single crowns
Stoichkov and Kirov [25]	65 patients, 65 implants	Retrospective study	Prosthesis height in mandibular molars ranged from 11 mm to 14 mm, and width from 5 mm to 8 mm. Higher dimensions linked to increased MBL.	Horizontal cantilevers up to 4 mm significantly increased MBL; vertical cantilevers up to 3 mm moderately increased MBL.	Higher MBL with C/I ratio >1.5/1	Significant linear correlation between C/I ratio and MBL	Higher C/I ratios in mandibular molars increase MBL compared to lower ratios

Lombardi et al. [21] used a multicenter prospective trial including 50 patients and 83 implants in order to raise generalizability. Malchiodi et al. [22] conducted a prospective cohort study with 136 patients and 259 implants, producing significant long-term data. Ramaglia et al. [23] conducted a longitudinal cohort study employing 78 implants in 34 mandibles and 44 maxillae in order to investigate extended time patterns. Rossi et al. [24] presented a prospective RCT with 45 patients and 60 implants. Stoichkov and Kirov [25] undertook a retroactive study with 65 patients and 65 implants, offering useful clinical insights.

Implant characteristics observed

Larger prosthesis dimensions (height 8-12 mm, width 5-8 mm) were connected to increased MBL, according to Anitua et al. [15], with horizontal cantilevers up to 3 mm considerably raising MBL and vertical cantilevers up to 2 mm having minimal impact. For short implants with an average prosthesis height of 10 mm and width of 6 mm, Bérczy et al. [16] showed that horizontal cantilevers up to 3 mm improved MBL, whereas vertical cantilevers had negligible impact. Particularly with horizontal cantilevers up to 4 mm greatly increasing MBL and vertical cantilevers up to 3 mm somewhat increasing MBL, Di Fiore et al. [17] showed higher crown heights (10-14 mm) were linked with increased MBL.

Fu et al. [18] observed that larger prosthetic dimensions, namely a height of 11 mm and a breadth of 6 mm, were associated with higher MBL. Moreover, vertical cantilevers up to 2 mm had no effect, whereas horizontal cantilevers up to 3 mm exhibited modest increases in MBL. While vertical cantilevers up to 2 mm had negligible impact, Hadzik et al. [19] found that horizontal cantilevers up to 3 mm affected both short and standard implants (short: height 9 mm, width 5 mm; standard: height 11 mm, width 6 mm), thereby increasing MBL. According to Lee et al. [20], increasing the dimensions of the prosthesis (height 9-13 mm, breadth 5-7 mm) generally resulted in reduced MBL. They also found that horizontal cantilevers up to 3 mm had a smaller influence on MBL, while vertical cantilevers up to 2 mm had a minimal effect.

Larger prosthesis dimensions (height 8-12 mm, width 4-6 mm) affected early MBL, according to Lombardi et al. [21], with horizontal cantilevers up to 3 mm enhancing early MBL and vertical cantilevers up to 2 mm having minimal effect. Malchiodi et al. [22] verified that surface-modified platform switching implants with greater prosthesis dimensions (height 10 mm, width 6 mm) were connected to increased MBL, although vertical cantilevers up to 2 mm had negligible impact and horizontal cantilevers up to 3 mm enhanced MBL.

Short implants with a prosthesis height of 9 mm and breadth of 4 mm exhibited higher MBL with bigger prosthesis dimensions, according to Ramaglia et al. [23]. Vertical cantilevers up to 2 mm showed no effect; horizontal cantilevers up to 3 mm raised MBL. Short implants (height 8 mm, width 5 mm) and conventional implants (height 10 mm, width 6 mm) showed higher MBL with bigger dimensions, Rossi et al. [24] noted. For short implants, horizontal cantilevers up to 3 mm greatly raised MBL; vertical cantilevers up to 2 mm had no effect. According to Stoichkov and Kirov [25], a higher MBL was correlated with prosthesis heights in mandibular molars (11-14 mm) and widths (5-8 mm). Vertical cantilevers up to 3 mm somewhat raised MBL; horizontal cantilevers up to 4 mm greatly increased MBL.

MBL and long-term impact observed

Anitua et al. [15] revealed a high correlation between the C/I ratio and both implant success and MBL surrounding the implant. Within particular C/I ratio ranges, the study found a great success rate. Bérczy et al. [16] reported that short implants displayed somewhat greater MBL than conventional ones. A larger C/I ratio and higher MBL showed a beneficial link, according to Di Fiore et al. [17]. MBL was discovered to be influenced by bone density and implant type, according to Fu et al. [18]. Hadzik et al. [19] noted identical MBL independent of the C/I ratio. Lee et al. [20] discovered that a higher MBL, especially in the upper jaw, was associated with a C/I ratio of ≤1. Lombardi et al. [21] observed that early bone loss was influenced by several elements and that bone loss changed with time. The C/I ratio was significantly correlated, according to Malchiodi et al. [22], between implant success and bone loss. Longer implants with lower C/I ratios were clearly shown to cause higher bone loss by Ramaglia et al. [23]. While Stoichkov and Kirov [25] showed increasing bone loss with higher C/I ratios, Rossi et al. [24] found more bone loss with short implants.

Discussion

Regarding the major influence of bigger prosthesis dimensions and horizontal cantilevers on MBL, Anitua et al. [15], Malchiodi et al. [22], and Di Fiore et al. [17] revealed comparable results. Short implants with appropriate cantilever management consistently showed similar success to conventional implants, according to Bérczy et al. [16], Hadzik et al. [19], and Ramaglia et al. [23]. Presenting more complex opinions on the impact of prosthetic dimensions, Fu et al. [18] and Lee et al. [20] highlighted other important considerations like bone density and site-specific effects. Early MBL management was underlined by Lombardi et al. [21] and Rossi et al. [24], as was the influence of prosthetic dimensions on long-term stability.

A good osseointegration depends on obtaining enough stability for the implant. Clinicians can maximize the timing of implant loading by closely assessing stability using objective and qualitative criteria. Combining resonance frequency analysis with insertion torque (IT) might provide a more accurate assessment of primary implant stability [26,27]. Although it is an easy and reasonably priced method to evaluate stability following implantation, it cannot evaluate secondary stability during bone remodeling [28-30].

Although there is continuous debate over the effects of implant diameter and length [31-33], there is agreement regarding the relevance of variables like bone quantity, density, and surgical technique [2,6,29-32]. While some research shows that surface characteristics and implant size have little effect on stability, others stress the need for implant length and bone structure [34,35]. Conversely, certain points of view hold that implant size rather than length influences stability [36].

Regarding the C/I ratio, our own study on the relevance of this ratio for implant success is in line with the results of Di Fiore et al. [37], implying that MBL is not much changed by a C/I ratio less than 2.2. Within a certain range (0.6/1 to 2.36/1), Garaicoa-Pazmiño et al. [38] discovered that lower MBL resulted from higher C/I ratios. This ending fits our results. Still, Garaicoa-Pazmiño et al. [38] presented a more exhaustive statistical analysis.

Padhye et al. [39] noted no clear change in MBL or implant longevity based on high and low C/I ratios; yet, our study revealed that increased MBL was associated with higher C/I ratios in particular scenarios (like the mandibular molar area). This suggests that other factors may have a greater influence on MBL and implant stability. The observation of little differences in MBL between small (<10 mm) and standard (≥10 mm) implants by Monje et al. [40] validates our conclusions, which show that short implants have similar MBL and long-term success relative to conventional implants. The results of Monje et al. [40] are therefore in perspective since our investigation also revealed a higher failure rate for short implants.

In terms of limitations, the retrospective nature of several studies could have brought prejudices into memory and choice. The several follow-up intervals could make regular evaluation of long-term findings more challenging. Furthermore, influencing the results could have been changes in the patient groups, clinical settings, and MBL testing techniques. Last but not least, even if it is vital, the focus on certain components, like the C/I ratio, may have hidden other vital components supporting implant stability and success. These shortcomings suggest that further standardized and regulated research is needed to validate and widen the conclusions.

## Conclusions

Greater MBL was found to be strongly correlated with bigger prosthetic dimensions, more specifically, with increased crown height and width. Particularly in the posterior areas, horizontal cantilevers often aggravated MBL; vertical cantilevers had quite little effect. Although they were more sensitive to prosthesis size and cantilever effects, short implants showed equivalent long-term success and MBL results to conventional implants when treated adequately. Often overshadowing the effects of primary implant stability, bone density, and IT became clear as important determinants of MBL. Early MBL was especially influenced by abutment height, mucosal thickness, and implant insertion depth; stabilization usually results in six months later. These results highlight the need for tailored treatment plans, including prosthesis dimensions and cantilever designs, to maximize implant success and longevity.
